# Supplementary material for: In vivo detection of optically-evoked opioid peptide release
Source: eLife. 2018 Sep 3;7:e36520. doi: 10.7554/eLife.36520 (PMC6135606; doi:10.7554/eLife.36520)
Supplement: Supplementary file 1. [file elife-36520-supp1.docx]

**Supplementary File 1: Opto-dialysis probe fabrication**

*Materials*

- Inlet = 40/100 (i.d./o.d.) fused silica capillary (5 cm)
- Outlet = 75/150 fused silica capillary (6 cm)
- Holder = 450/670 fused silica capillary (5 mm)
- Inlet cover = 150/360 fused silica capillary (8 mm), covered with 30G thin wall Teflon tubing (7mm)
- Outlet cover = 180/360 fused silica capillary (10 mm), covered with 30G thin wall Teflon tubing (9 mm)
- Microdialysis semi-permeable membrane (material and pore size dependent on peptides analyzed, we used a 60 kDa molecular weight cutoff, polyacrylonitrile membrane with a slight negative charge (AN69, Hospal, Bologna Italy))
- Fiber optic (total length 14 mm) with attached ferrule, this can be adjusted to appropriate length depending on region of interest. (See citations in main text)
- Glue: Thin cyanoacrylate glue, gel superglue, five minute epoxy, glue accelerator

*Instructions*

1. Place the inlet and outlet capillaries side-by-side and slide the outlet capillary down equal to the length of the desired active area (i.e. if you want to make a 1 mm probe, offset the outlet 1mm below the inlet).
2. Using a needle, apply thin liquid glue to adhere capillaries together. Only apply the least amount of glue needed
3. Carefully slide a piece of microdialysis membrane over both the inlet and outlet, avoiding pinching and kinking of membrane. Cut excess membrane off. Cutting the membrane at a 45° angle can help if it is problematic to slide over both capillaries
4. Mix a small amount of 5-minute epoxy, and let slightly harden to a consistency that is able to hold stiff peaks. Once the correct consistency is achieved, use a clean hypodermic needle and carefully fill the end of the membrane to create a complete glue plug. Note: If you use the epoxy too early you will get a large glue plug. If you use the epoxy too late, it will be too stiff and not fill the tip of the membrane completely, leaving you with a hole for perfusion media to leak out of. Be careful not to get epoxy on the exterior of the membrane. Let the glue plug dry for 2-3 h before proceeding.
5. Once the glue plug is dry, gently tap/move the membrane down the capillary until the inlet is ~100 μm away from the glue plug.
6. Mix a small amount of 5-minute epoxy and carefully apply a small amount around the entire backside of the membrane. You should see the glue fill the back. Let dry overnight. If you apply too much, the glue can wick up the membrane and clog the outlet. If you are careful you can create an even seal around the entire membrane
7. Slide the 450/670 capillary holder over the back side of the inlet/outlet assembly, followed by sliding the fiber optic into the backside of the capillary holder. The fiber optic is rigid and cannot handle much angled force.
8. Position the fiber optic so the tip is adjacent to the outlet, keeping the capillary holder is as close as possible to the ferrule.
9. Carefully apply the thin cyanoacrylate glue to the secure the capillary holder to the inlet/outlet assembly and fiber optic together. Careful not to apply too much glue.
10. Take 30G thin walled Teflon tubing and slide over the inlet and outlet cover capillaries until all but ~ 1mm of capillary is covered.
11. Use a sharp razor blade and gently score the Teflon closest to the exposed capillary. This scoring will help glue to hold Teflon in place.
12. Slide the inlet and outlet covers over the respective inlet and outlet and secure with thin cyanoacrylate (applied to the joint side/exposed capillary side of inlet/outlet cover)
13. Use a large plastic pipette tip, and cut a small piece off the pipette tip to use as a junction cover.
14. Use gel glue and apply around the entire joint.
15. Carefully slide the pipette tip over the membrane/fiber optic and up to the joint with glue.
16. Apply some glue accelerator to the joint. Let the joint dry, for a few minutes and reinforce with 5-minute epoxy around the inlet/outlet covers and fiber optic.
17. Cut excess inlet and outlet capillaries as close to the inlet and outlet covers right before use.
