# Supplementary material for: In vivo detection of optically-evoked opioid peptide release
Source: eLife. 2018 Sep 3;7:e36520. doi: 10.7554/eLife.36520 (PMC6135606; doi:10.7554/eLife.36520)
Supplement: Transparent reporting form [file elife-36520-transrepform.pdf]

eLIFE
1st Floor
24 Hills Road
Cambridge CB2 1JP, UK
P 01223 855340
W elifesciences.org
T @elife

# *eLife's* transparent reporting form

We encourage authors to provide detailed information *within their submission* to facilitate the interpretation and replication of experiments. Authors can upload supporting documentation to indicate the use of appropriate reporting guidelines for health-related research (see EQUATOR Network), life science research (see the BioSharing Information Resource), or the ARRIVE guidelines for reporting work involving animal research. Where applicable, authors should refer to any relevant reporting standards documents in this form.

If you have any questions, please consult our Journal Policies and/or contact us: editorial@elifesciences.org.

**Sample-size estimation**

- You should state whether an appropriate sample size was computed when the study was being designed
- You should state the statistical method of sample size computation and any required assumptions
- If no explicit power analysis was used, you should describe how you decided what sample (replicate) size (number) to use

Please outline where this information can be found within the submission (e.g., sections or figure legends), or explain why this information doesn't apply to your submission:

No explicit power analysis was used in advance of the study, but we made every effort to use groups of mice similarly powered to previous and related studies (Patterson et al, Endocrinology, 2015; Al-Hasani et al, Neuron, 2015

**Replicates**

- You should report how often each experiment was performed
- You should include a definition of biological versus technical replication
- The data obtained should be provided and sufficient information should be provided to indicate the number of independent biological and/or technical replicates
- If you encountered any outliers, you should describe how these were handled
- Criteria for exclusion/inclusion of data should be clearly stated
- High-throughput sequence data should be uploaded before submission, with a private link for reviewers provided (these are available from both GEO and ArrayExpress)

Please outline where this information can be found within the submission (e.g., sections or figure legends), or explain why this information doesn't apply to your submission:

eLife Sciences Publications, Ltd is a limited liability non-profit non-stock corporation incorporated in the State of Delaware, USA, with company number 5030732, and is registered in the UK with company number FC030576 and branch number BR015634 at the address 1st Floor, 24 Hills Road, Cambridge CB2 1JP | August 2014

Every effort was taken to ensure both technical (verification of opioid detection and probe integrity *in vitro* using LC-MS) and biological (collection of dialysate from freely moving mice and analysis using LC-MS) replication.
Technical:
* Thorough validation of isotopically labeled dynorphin internal standard ensuring no interference with endogenous peptide detection and improving quantification, page 5, lines 61-76, Figure 1 (Four replicates per sample, figure 1 legend).
* Tested our custom-made probe prior to fabricating more for animal implantation to ensure measurable changes in peptide stock concentrations, page 6, 86-90, Figure 2 (Four probes, figure 2 legend).
Biological:
*Each biological replicate was a new mouse
*Correct regional expression of the AAV5-DIO-ChR2-eYFP in the brain was verified in addition to placement of the opto-dialysis probe in either the vNAcSh or dNAcSh, which are represented on hit maps in supplemental figure 3 and pages 14-15, lines 307-318. We used this as our exclusion criteria.
* To ensure accurate detection in each animal, we only included animals that had average basal levels above the limits of detection. Page 13-14, lines 281-287
* As a positive control for detecting dynamic changes *in vivo* by nLC-MS and to establish sampled neuron responsivity to stimuli, we infused 100 mM $K^+$ aCSF at the end of each collection experiment. Page 6, line 100-107 and Supplemental figure 2

eLife Sciences Publications, Ltd is a limited liability non-profit non-stock corporation incorporated in the State of Delaware, USA, with company number 5030732, and is registered in the UK with company number FC030576 and branch number BR015634 at the address 1st Floor, 24 Hills Road, Cambridge CB2 1JP | August 2014

**Statistical reporting**

- Statistical analysis methods should be described and justified
- Raw data should be presented in figures whenever informative to do so (typically when N per group is less than 10)
- For each experiment, you should identify the statistical tests used, exact values of N, definitions of center, methods of multiple test correction, and dispersion and precision measures (e.g., mean, median, SD, SEM, confidence intervals; and, for the major substantive results, a measure of effect size (e.g., Pearson's r, Cohen's d)
- Report exact p-values wherever possible alongside the summary statistics and 95% confidence intervals. These should be reported for all key questions and not only when the p-value is less than 0.05.

Please outline where this information can be found within the submission (e.g., sections or figure legends), or explain why this information doesn't apply to your submission:

The University of Michigan Center for Statistical Consultation and Research helped design a linear mixed model analysis appropriate for the optogenetic-dialysis study using SPSS Statistics software. The linear mixed model analysis was chosen to account for variations within and between mice and to account for missing data points within individual animals following sample loss or mechanical failure of the instrument. The linear mixed model was used to determine differences in basal conditions, effect of photostimulation relative to basal conditions, and prolonged effects after photostimulation relative to basal conditions. Linear mixed models were used to compare between genotypes within each region sampled, and between regions within *dyn-Cre* + and *dyn-Cre* – genotypes. In all cases, significance was defined as $p \leq 0.05$. Page 15, lines 320-330.

Each statistical test, n number, statistical result and p-values are provided in the appropriate figure legends (Figures 1, 2, 3)

(For large datasets, or papers with a very large number of statistical tests, you may upload a single table file with tests, Ns, etc., with reference to sections in the manuscript.)

**Group allocation**

- Indicate how samples were allocated into experimental groups (in the case of clinical studies, please specify allocation to treatment method); if randomization was used, please also state if restricted randomization was applied
- Indicate if masking was used during group allocation, data collection and/or data analysis

Please outline where this information can be found within the submission (e.g., sections or figure legends), or explain why this information doesn't apply to your submission:

The groups compared were the genotypes (dynorphin cre+ vs Dynorphin cre-) and 2 subregions (dorsal and ventral) of the Nucleus accumbens shell. When collecting dialysate and analyzing via LC-MS the experimenter was blinded to the genotype and region. Page 4-5, lines 45-55. Figure 3.

**Additional data files ("source data")**

- We encourage you to upload relevant additional data files, such as numerical data that are represented as a graph in a figure, or as a summary table
- Where provided, these should be in the most useful format, and they can be uploaded as "Source data" files linked to a main figure or table

- Include model definition files including the full list of parameters used
- Include code used for data analysis (e.g., R, MatLab)
- Avoid stating that data files are "available upon request"

Please indicate the figures or tables for which source data files have been provided:
